# A Closer Look at Corticothalamic “Loops”

**DOI:** 10.3389/fncir.2021.632668

**Published:** 2021-02-02

**Authors:** Kathleen S. Rockland

**Affiliations:** Department of Anatomy & Neurobiology, Boston University School of Medicine, Boston, MA, United States

**Keywords:** corticogeniculate, heterogeneity, layer 6, reciprocity, cortical cell types

A defining feature of primary sensory cortical areas has been that they receive and reciprocate thalamic “relays” of external events. The interactions of sensory input, autonomous cortical processes, and more cognitive functions, however, remain under active investigation; and continuing work is bringing into better focus the fuller spatial, temporal, and dynamic complexity of the cortical-subcortical sensory pathways, conveniently described as “loops.” There are reports that corticothalamic (CT) connections regulate the mode of thalamic activity and can exert a flexible control of thalamocortical (TC) inputs (mouse cortex: Mease et al., 2014; Crandall et al., 2015; Guo et al., 2017; Kirchgessner et al., 2020). Auditory CT feedback to the medial geniculate body is found to contribute to the detection of harmonicity, an important grouping cue in the perception of complex sounds (ferrets: Homma et al., 2017). Arousal related modulation can already be demonstrated at retinal inputs to the thalamus (mouse: Liang et al., 2020); and brainwide recordings of multiple structures suggest “an ubiquitous mixing of sensory and motor information,” happening as early as primary sensory cortex (mouse: Stringer et al., 2019).

In this Opinion, I briefly bring together several anatomical features of CT connections, relevant to an emerging view of cognitive-sensory processes; namely, (1) cell type diversity, which may only partly be related to segregated parallel processing; (2) reciprocity, which may not be monosynaptic cell-to-cell and may not be strictly topographic; and (3) the massive convergence of multiple intrinsic cortical and multiple direct and indirect extrinsic connections beyond the primary thalamus. This Topic asks: do feedback circuits perform common functions, and are feedback mechanisms unique or shared across sensory modalities? Overall, the evidence supports a multilevel diversity; and I would like to propose that this diversity, not unlike that of cell types (e.g., Cembrowski and Spruston, 2019), is central to brain organization and connectivity, and thus an important perspective in the context of cortico-subcortical sensory processing (CT projections from layers 5 or 6 to higher order thalamic nuclei will not be covered here; but see discussion and references in e.g., Rockland, 2019; Usrey and Sherman, 2019; Vanni et al., 2020).

## Ct Projection Neurons: Layer 6

Across species and across modalities, CT projections to primary sensory thalamus originate from excitatory neurons in layer 6. However, the proportion of CT neurons within layer 6 is variable by species. Corticogeniculate (CG) neurons are reported to comprise about 15% in primates but 50% in carnivores [reviewed in Hasse and Briggs (2017), Vanni et al. (2020)]. Whether this proportion also varies within an area (e.g., foveal vs. peripheral visual field for area V1) is not known.

CT neurons in layer 6 are heterogeneous according to multiple criteria (Baker et al., 2018). First, physiological recordings have established that stimulus driven conduction times are variable, ranging from short to extremely long (<2 to 40–50 ms), probably due to presence or absence of myelin [reviewed in Hasse and Briggs (2017), Stoelzel et al. (2017)]. In rabbit visual cortex, fast CG neurons are preferentially situated in the superficial stratum of layer 6 (Stoelzel et al., 2017). Of the CG neurons characterized as slow, “many of the slowest are silent and [sensory stimulus] unresponsive,” perhaps arguing for a multimodal or more complex role (or, “plastic reduction,” Stoelzel et al., 2017).

Second, multiple groups of CT neurons have been distinguished on the basis of apical dendritic morphology. For macaque visual cortex (V1 and the smaller population in V2), broad categories of CG neurons are distinguished on the basis of long or short apical dendrites, respectively, extending toward layer 1 or terminating in layer 3 (Hasse and Briggs, 2017; or two classes, with subtypes: Wiser and Callaway, 1996). Unlike pyramidal neurons in layers 5, which typically extend to and ramify in layer 1, the computationally significant apical tuft is often absent or poorly developed (Ledergerber and Larkum, 2010; Thomson, 2010; Baker et al., 2018).

Third, in macaque primary visual cortex, six main functional clusters have been reported based on responsiveness to visual stimuli (i.e., achromatic gratings, with varying parameters; Hawken et al., 2020). Transcriptomic identity was not determined, nor the actual connectional identity of the neurons (i.e., whether these project to the LGN, or the claustrum, or are solely intrinsic); but these data might be expected from continuing work, as in other reports in mouse (e.g., Tasic et al., 2018; Cembrowski and Spruston, 2019).

A fourth differentiating factor is the number, identity, and arrangement of inputs to individual CT neurons. Complete input maps are so far not available at the single neuron level; but the conspicuous difference in apical dendritic extent clearly implies differences of input number and identity, and thus in the integrative capacity of the individual neurons. TC inputs, for example, can contact CT neurons directly in layer 4 (on apical dendrites) and/or in layer 6 (on basal dendrites), in addition to the polysynaptic input from cortical neurons in layer 4. In the macaque, layer 6 inputs can occur as collaterals of TC axons continuing to layer 4 (area V1: Blasdel and Lund, 1983; Freund et al., 1989; somatosensory cortex: Garraghty and Sur, 1990). In the rodent, direct layer 6 terminations, although less dense than those to layer 4, have been demonstrated in rat barrel cortex (Constantinople and Bruno, 2013; Crandall et al., 2017), where the postsynaptic targets have been identified (proportion unknown) as neurons in layer 5B, corticocortical neurons (CC) in layer 6, and CT neurons in layer 6 (Constantinople and Bruno, 2013). The latter receive only weak direct inputs from VPm (Crandall et al., 2017).

Other inputs to CT neurons are more numerous than the TC inputs and include a range of intrinsic excitatory, intrinsic inhibitory, and some excitatory cortical feedback inputs (to layer 6 or, in macaque visual cortex, layer 4B for the neurons with longer apical dendrites). Of these, several potentially convey multi-sensory information. Macaque V1 receives input from auditory (Falchier et al., 2002; Rockland and Ojima, 2003) and parietal association areas (Borra and Rockland, 2011), particularly in the representation of the peripheral visual field. In the mouse area V1, layer 6 neurons receive a widespread head-motion signal from retrosplenial cortex (Velez-Fort et al., 2018).

Fifth, CT neurons in layer 6 may receive differential neuromodulatory inputs. These are known to have multiple effects, which in part depend on receptor distributions and substance concentration, likely to vary across neurons. Acetylcholine, for example, preferentially contributes to a facilitation of CT layer 6 neurons by an interaction of muscarinic and nicotinic receptors (in rats: Yang et al., 2020; and for recent reviews of neuromodulators: Coppola and Disney, 2018; Jacob and Nienborg, 2018; Radnikow and Feldmeyer, 2018). In primary somatosensory and visual cortices (but also non-primary sensory areas), orexin, a peptide associated with wakefulness and attention, excites cortical neurons in layer 6B by a direct postsynaptic action (rats: Bayer et al., 2004).

A common interpretation of CT neuron subtypes has been that these are related to segregated, parallel submodality processing. Transcriptional results, however, are tending to support a continuous variation and more textured heterogeneity within cell types as classically defined (Cembrowski and Menon, 2018; Cembrowski and Spruston, 2019). In mouse barrel cortex, for example, single gene expression profiles are preferentially associated with the two broad classes of layer 6 CT neurons, as defined by distinctive targeting by VPm alone or by VPm and POm (Chevee et al., 2018). Further, altering the neuronal activity state, by partial removal of whiskers, not only resulted in asynchronous gene expression between the two subtypes, but also in variation among neurons of the same subtype. This was discussed as a byproduct of regulatory redundancy, a temporal snapshot of dynamic processes, or as an inherent molecular variability in some ways essential to population-level function [Dueck et al., 2015, in Chevee et al. (2018)].

## Ct and Tc Connections Are Only Approximately Reciprocal

Reciprocity of CT and TC connections is basic to the idea of a recursive loop; and zonal topographic reciprocity has been repeatedly demonstrated, by target and origin correspondence of cortical and thalamic projections. Visualization of single CG axons in cats, however, demonstrates a finer organization, consisting of a central region of dense terminations (400–500 μm across) and a larger, sparser surround zone (maximum spread of 500–1,500 μm; *n* = 14 axons, in cat: Murphy and Sillito, 1996). The smaller central region is consistent with a retinotopic correspondence; but the larger surround implies more global, not necessarily retinotopic processes, perhaps related to stimulus context over longer distances (Murphy and Sillito, 1996; and related, Darian-Smith et al., 1999). Comparably detailed data are lacking for other species and modalities, although whole brain imaging offers a promising new resource for investigating these questions (in rodent: Winnubst et al., 2019).

Does reciprocity apply at the single cellular level (“looped” reciprocity)? Potentially, the several TC neurons postsynaptic to a layer 6 CT neuron could contact the same CT neuron, by direct monosynaptic terminations in layer 6 and/or indirect polysynaptic input via terminations in layer 4. If monosynaptic reciprocity actually occurs is unknown, but if so, it would be only a small percentage of the total synaptic input for the respective cortical and thalamic neurons. As reference, we can consider what is known of convergence and divergence of retinogeniculate axons, where a given LGN neuron (in cat) is estimated to receive input from 10 or more retinal axons, and each retinal axon diverges to innervate more than 20 relay thalamic cells. For four thalamic relay neurons, the proportion of retinal input from a single identified retinogeniculate axon varied from 2 to 100% [reviewed in Bickford (2019), and related Guo et al. (2020)].

For the more experimentally accessible visual cortical connections, information about global contours in a cluttered background is reported to emerge initially in “upstream” area V4 and only 40 ms later in V1, and then to develop in parallel in both areas. This has been interpreted as an incremental mechanism, drawing on both bottom-up and reentrant processes (Chen et al., 2014).

## Divergence/Convergence

CT and TC connections are often discussed as if a segregated two-way (“loop”) network. CT layer 6 neurons in the sensory areas, however, project not only to thalamus, but also to the reticular nucleus (RT), source of inhibitory inputs to the same thalamic nucleus (and potentially to the same, cortically-recipient thalamic neurons?). This connectivity motif is all the more compelling, since layer 5 CT projections (to association thalamus) tend to avoid giving collaterals to RT (e.g., Usrey and Sherman, 2019). The quantitative and spatial synaptic relationships have not been investigated in detail; and the degree of variability across CT neurons is unknown as concerns synaptic numbers and distribution. Recent results demonstrate that many, but not all the RT neurons exert a graded, frequency dependent inhibition of LGN relay cells (in mice: Campbell et al., 2020).

In the macaque visual system, secondary (i.e., less dense) CT projections originate from layer 6 of extrastriate area V2 (Briggs et al., 2016). It is currently unknown whether these extrastriate CT terminations converge on the same thalamic neurons as the denser projections from V1, or whether these CT neurons in V2 have feedback branches to V1 and/or to the pulvinar. There are of course multiple potential polysynaptic interconnections across the striatum, colliculus, pulvinar, and claustrum with both sensory thalamus and sensory cortex (“recurrent and highly interactive”: Kravitz et al., 2013, for visual pathways).

The action of CT projections is not necessarily uniform across a thalamic nucleus (as, source and target specificity). Sensory thalamic nuclei are not homogeneous, but have nucleus-specific terminal configurations. In the LGN, for example, synaptic organization differs in encapsulated (glomerular) vs. interstitial zones, each having a differential juxtaposition of retinogeniculate, CG terminations, and local inhibitory neurons in relation to TC relay cell dendrites. The central visual representation of the LGN contains more encapsulated zones than regions receiving input from the peripheral retina. CT synapses (and inhibitory synapses from the reticular nucleus) predominate in the non-encapsulated, interstitial zones (Bickford, 2016, 2019). Here, also, talking about a two-way “loop” overly simplifies, and thereby limits progress.

## Common Sensory Cortical Calculation?

The classical anatomical terminology recognized primary sensory cortices as “heterotypical,” with specialized architectonic features. Thus, area V1 in the visually dominant macaque has an elaborate laminar and modular organization; and in mice and rats, there is the intricate barrel/septum architecture in the somatosensory cortex. Intra-modal architectural features, however, differ even across related species (layer 4A in humans and non-human primates: Preuss and Coleman, 2002) and across individuals (size and number of ocular dominance columns in macaque: Horton and Hocking, 1996). The basic pattern of CT connections is recognizable across modalities and, with variations, phylogenetically conserved (summarized for rodents, cats, and monkeys: Rouiller and Welker, 2000); but it is not stereotyped: functionally significant quantitative parameters and microcircuitry vary by area and species. From the perspective of anatomical circuitry and architectural specializations, the idea of “common cortical calculation” seems hard to endorse.

In summary, the characterization of CT connections as “loops” is best seen as provisional. Both conceptually and methodologically, research is moving rapidly to a finer granularity and multi-dimensionality, taking account of subtypes of CT neurons, diversity of microcircuits, a plurality of physiological effects, and abundant and intricate interactions across systems, including neuromodulatory (i.e., the loop is multi-stranded). CT connections are not an isolated silo, but are components of large interacting networks (confluence of loops). This is in no way a new observation (Tantirigama et al., 2020 among others), but worth repeating, especially since the technical tools now available offer new opportunities for probing activity dependent interactions over whole brain networks, an essential approach for further investigations of basic elements of CT processing.

## Author Contributions

KR is responsible for content and presentation.

## Conflict of Interest

The author declares that the research was conducted in the absence of any commercial or financial relationships that could be construed as a potential conflict of interest.
